# Complete genome sequences of six *Serratia* strains with multiple resistance genes isolated from food products in Canada

**DOI:** 10.1128/mra.00301-25

**Published:** 2025-06-23

**Authors:** Mingsong Kang, Darrell Harjadi, Emily Hoover, Hanhong Dan, Bailie Adam, Hongsheng Huang

**Affiliations:** 1 Ottawa Laboratory – Fallowfield, Canadian Food Inspection Agency 5737, Ottawa, Ontario, Canada; 2 Greater Toronto Area Laboratory, Canadian Food Inspection Agency 5737, Toronto, Ontario, Canada; University of Maryland School of Medicine, Baltimore, Maryland, USA

**Keywords:** *Serratia* species, *Serratia liquefaciens*, *Serratia fonticola*, *Serratia nevei*, whole-genome sequences, food-borne pathogens, Canada

## Abstract

*Serratia* species are widespread, opportunistic Gram-negative microbes found in diverse environments, with some species posing risks to humans, potentially through food. In this announcement, we present six complete genome sequences of two strains for each of *S. liquefaciens*, *S. fonticola*, and *S. nevei,* respectively, isolated from food in Canada.

## ANNOUNCEMENT

*Serratia* species, members of the *Enterobacteriaceae* family, are Gram-negative bacteria capable of causing opportunistic infections. They thrive in diverse environments, including food, water, and animal hosts (1–4). Species such as *S. liquefaciens*, *S. nevei,* and *S. fonticola* are increasingly recognized as human pathogens (4–7). This report presents the complete genome sequences of two strains each of *S. liquefaciens*, *S. fonticola,* and *S. nevei*, isolated from food samples in Canada in 2013 and 2016. Pure colonies were isolated by diluting homogenized food samples into buffered peptone water (1:9 ratio), repeated streaking on Tryptic Soy Agar (TSA), and incubating for approximately 16–20 hours at 35 °C. Initial taxonomic identification was performed using the VITEK MS system (bioMérieux) and subsequently confirmed by sequencing, with species-level differences detailed in Table 1.

**TABLE 1:** Sequencing parameters and genes for virulence, antibiotics, heavy metal, and acid resistance

Strain ID (GTA-)	Species^*b*^	Food source and location	SR^*a*^			LR^*a*^				Total length (M)	GC (%)	Protein count	Plasmids	Genes^*a*^				Accession number	
			No. of reads pre-filtered (M)^*c*^	No. of reads post-filtered (M)^*c*^	Coverage(x)	No. of reads pre-filtered^*d*^	No. of reads post-filtered^*d*^	N_50_ (kb)^*d*^	Coverage (×)					VF	AMR	MR	AR	GenBank	SRA
SR02	*S. liquefaciens*	Beef (Ontario)	6.93	5.48	164	216,358	162,819	17.3	287	5.54	55.1	5,091	3	1,256	5	1	2	GCA_048537415.1	SRX27876135 SRX27876134
SR06	*S. fonticola*	Romaine (Quebec)	2.80	1.60	48	251,548	203,432	24.3	426	5.95	53.7	5,371	1	1,431	3	6	2	GCA_048571845.1	SRX27876139 SRX27876138
SR07	*S. fonticola*	Spinach (Nova Scotia)	4.90	4.59	124	132,306	102,683	23.1	199	5.73	53.7	5,080	2	1,392	3	5	2	GCA_048537545.1	SRX27876141 SRX27876140
SR09	*S. liquefaciens*	Pear (Ontario)	2.53	1.76	59	333,784	210,603	19.1	381	5.34	55.2	4,852	0	1,217	5	3	2	GCA_048537535.1	SRX27876143 SRX27876142
SR10	*S. nevei*	Plum (Ontario)	3.13	1.97	57	300,111	244,298	19.7	507	5.48	58.9	5,048	0	1,223	7	20	2	GCA_048849215.1	SRX27876145 SRX27876144
SR11	*S. nevei*	Peach (Quebec)	2.65	1.70	55	336,873	261,028	16.0	455	5.33	59.7	4,946	0	1,239	8	1	2	GCA_048571855.1	SRX27876137 SRX27876136

^*a*^ SR: short reads; LR: long reads; VF: virulence factor; AMR: antimicrobial resistance; MR: metal resistance; AR: acid resistance.

^*b*^ Species were identified using GTDB-TK within the NanoPhase pipeline and further confirmed by NCBI’s taxonomy check. VITEK MS system misidentified SR10 AND SR11 as *S. marcescens* due to the lack of a database of *S. nevei*.

^*c*^ Data obtained from the Fastp pipeline.

^*d*^ Data obtained from the NanoComp pipeline.

Genomic DNA from the isolates was extracted from 18 hours of cultures at 37 °C in Tryptic Soy Broth, grown from single colonies, using either PureLink Genomic DNA Mini Kit (Invitrogen, US) or NanoBind CBB Kit (PacBio, US). Illumina sequencing libraries were prepared with the Illumina DNA Prep Kit (Illumina, US) and sequenced on the MiSeq platform with the MiSeq reagent kit v3 (Illumina, US), generating 11,476,016 paired-end reads (300 bp), which were filtered and trimmed using Fastp version 0.23.2 (8). For nanopore sequencing, the libraries were prepared with the SQK-NBD114.24 ligation barcoding kit without shearing and sequenced on a MinION Mk1B device with an FLO-MIN114 flow cell. After base-calling and trimming using Dorado version 0.9.1 with the sup model, 1,570,980 reads were obtained. Subsequently, reads shorter than 600 bp and with a quality score below 15 were filtered using NanoFilt version 2.8.0 (9). NanoComp version 1.23.1 was utilized to assess long-read quality pre- and post-filtering (9). Genome assembly, polishing, quality assessment, and bacterial classification (GTDB-TK v.2.3.2 with reference data version r220) were performed using an all-in-one pipeline NanoPhase v.0.2.3 with isolate module: hybrid strategy (10). Genome circularity and rotation using *dnaA* as the starting point was determined with Circlator v1.5.5 (11). Mean coverage depth was obtained from log files of Flye v2.9.2 (long reads) (12) and Polypolish v0.7.17 (short reads) (13) within the Nanophase pipeline. Gene predictions and annotations were performed using NCBI Prokaryotic Genome Annotation Pipeline v6.9 (14). Resistance genes (metal, acid, and antimicrobial resistance [AMR]) were identified using AMRFinderPlus v4.0.19 with database v2024-12-18.1 (15), virulence factor genes predicted by PathoFact v1.0 (16), and plasmids identified by PlasmidHunter v1.4 (17). Default parameters were used unless otherwise specified.

Table 1 presents the short-read and long-read sequencing data. Genome assemblies average 5.56 Mbp in length, with GC content ranging from 53.7% to 59.7%. Gene and protein counts vary by strains. The presence of plasmids and numbers of virulence factors (VF), antimicrobial resistance (AMR), mobile resistance (MR), and acquired resistance (AR) genes reflect genetic diversity among isolates (Table 1).

## Data Availability

Genome sequences and raw sequencing data are available at the NCBI under the accession numbers listed in Table 1.
